# A Historical Reassessment of the Authorship Year of *Brachyteles arachnoides* (Primates: Atelidae)

**DOI:** 10.1002/ajp.70064

**Published:** 2025-08-13

**Authors:** José E. Serrano‐Villavicencio, Joyce R. Prado

**Affiliations:** ^1^ Mamíferos, Museu de Zoologia Universidade de São Paulo São Paulo Brazil

**Keywords:** Atelinae, Muriqui, nomenclature, taxonomic revision, type specimen

## Abstract

The authorship of *Brachyteles arachnoides* has traditionally been ascribed to É. Geoffroy Saint‐Hilaire, 1806. However, É. Geoffroy Saint‐Hilaire's original description was based entirely on secondary accounts, namely, Browne's (1756) *Simia 2* and Edwards' (1764) report of a brown, long‐limbed, and four‐fingered monkey, without directly examining specimens or illustrations. Browne's *Simia 2* describes a large brown primate with a prehensile tail and four‐fingered hands in Jamaica, characteristics that could apply to either *Ateles* or certain *Brachyteles* populations. Edwards' account, meanwhile, references two four‐fingered “spider monkeys” observed in London but lacks sufficient detail for definitive taxonomic assignment. Historical trade data further undermine this link, as 18th‐century Jamaica likely hosted Colombian/Panamanian primates, with no evidence of Brazilian primate imports. Étienne Geoffroy Saint‐Hilaire obtained the first verifiable *Brachyteles* specimen only in 1808, seized during Napoleon's Lisbon campaign. His 1809 redescription, including an illustration and the specimen MNHN‐ZM‐2007‐1475, meets modern taxonomic standards, whereas the 1806 name, based solely on ambiguous accounts, fails ICZN criteria for type association. We argue that *Ateles arachnoides* É. Geoffroy Saint‐Hilaire, 1806, constitutes a *nomen dubium*, as it cannot be tied to verifiable material. Instead, we validate *Ateles arachnoides* É. Geoffroy Saint‐Hilaire, 1809, with MNHN‐ZM‐2007‐1475 as the holotype by monotypy. This redefinition stabilizes the species' nomenclature, anchoring it to a concrete specimen and Geoffroy Saint‐Hilaire's empirically grounded 1809 work. By resolving these historical ambiguities, we provide a clearer framework for understanding *Brachyteles* taxonomy and highlight the importance of type specimens in early primatological classifications.

## Summary

- The conventional attribution of *Brachyteles arachnoides* to Étienne Geoffroy Saint‐Hilaire (1806) is contested, as the original description relied solely on unverifiable secondary accounts and lacked direct examination of the specimens.
- Historical evidence suggests that Browne's *Simia 2* and Edwards' “brown monkey” lack links to *Brachyteles*, likely referring to *Ateles* species from the Caribbean region, given the historical trade routes between Jamaica and mainland Central and South America.
- *Ateles arachnoides* É. Geoffroy Saint‐Hilaire, 1809 is recognized as the correct authorship date of the Southern Muriqui, with MNHN‐ZM‐2007‐1475 designated as the holotype by monotypy, supported by a detailed description and illustration from 1809.

## Graphical abstract

Key historical milestones in the taxonomy of *Brachyteles arachnoides*. It begins with Browne's (1756) early depiction of *Simia 2*, “The Four‐fingered Monkey”, in Jamaica, followed by Edwards' (1764) account of a brown four‐fingered monkey in the streets of London. In 1806, Étienne Geoffroy Saint‐Hilaire named *Ateles arachnoides* based on these accounts. The classification was later refined after he examined a specimen from the Museu de Ajuda in 1808, culminating in a redescription in 1809.

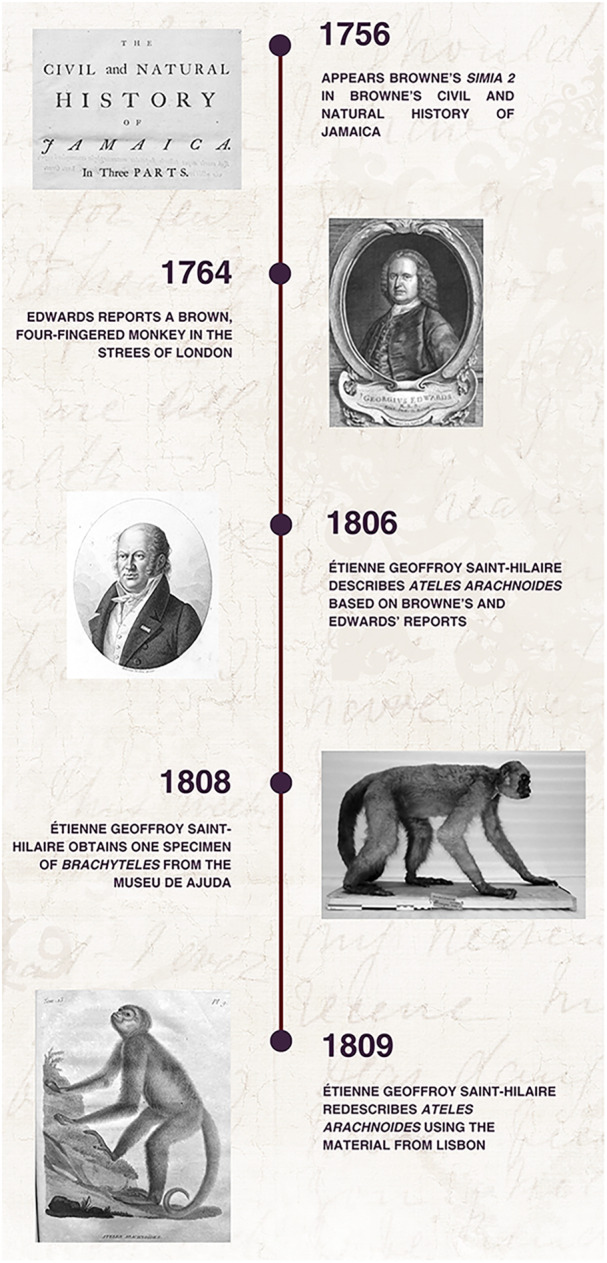

The authorship year of the Southern Muriqui, *Brachyteles arachnoides*, has been widely accepted as 1806. At the time, however, É. Geoffroy Saint‐Hilaire did not have the opportunity to examine a species specimen. His description relied entirely on secondary sources, as he had evidently never seen a living specimen, a preserved individual, or even an illustration. Consequently, É. Geoffroy Saint‐Hilaire's description (1806) was based solely on two references: Browne (1756) and Edwards (1764). For this reason, we first examine whether the primates mentioned in Geoffroy Saint‐Hilaire's two cited references can be reliably associated with *Brachyteles* material.

The first reference cited by É. Geoffroy Saint‐Hilaire (1806) is Browne's (1756) *The Civil and Natural History of Jamaica*. In Section IV of this study, Browne (1756: 489) reported the “Anthropomorphits or such as partake more or less of human shape and disposition,” which included four primates, Simia 1, 2, 3, and 4. É. Geoffroy Saint‐Hilaire (1806) cited this “*Simia 2*”, which Browne identifies as “The Four‐fingered Monkey” and provides a brief Latin description: *“Fusca major, palmis tetradactylis, caudâ præhensili ad apicem subtus nudâ”*, which translates to “Large brown, with four‐fingered hands, tail prehensile at the apex, bare beneath”. This description emphasizes the primate's distinctive morphological traits, particularly the presence of a tactile pad on the underside of the distal portion of its prehensile tail—a synapomorphy characteristic of the Atelidae family, and the presence of only four fingers in its hands, only observable in *Ateles* and some populations of *Brachyteles*.

Browne further notes that this primate originated from the mainland and was consumed by indigenous populations. These details remain unchanged in the second edition of Browne's work, published in 1789, even though Browne himself was not involved in its production (Hutcheon 2023). Browne's *Simia 2* was referenced in Linnaeus (1758) *Systema Naturae*, in the account of [*Simia*] *paniscus* (species 7, currently a synonym of *Ateles paniscus*). Additionally, Linnaeus (1758: 26) reproduced *Simia 2*'s Latin description verbatim into the synonymy of *S. paniscus*, which was maintained in the subsequent editions of *Systema Naturae*.

The second reference used by É. Geoffroy Saint‐Hilaire (1806) was Edwards (1764: 222), who reported two four‐fingered, long‐limbed monkeys. The first was a Black Monkey, known as a Spider Monkey, while the second was described as “in all respects like the former” but with brown hair. Regrettably, Edwards provides no additional information or illustrations of these species, leaving further clarification elusive. Edwards' works were largely used by Linnaeus in several editions of his *Systema Naturae*, mostly in the part of birds in which Edwards' contributions were monumental (Robson 1776).

Regarding primates, Edwards documented several species in his works *A Natural History of Uncommon Birds* (Edwards 1743, 1747, 1750, 1751) and its successor, *Gleanings of Natural History* (Edwards 1758, 1760, 1764). In these publications, he described various species of lemurs, as well as primates from Asia, Africa, and the Americas. However, in all editions of *Systema Naturae* published after Edwards's works, only four American primates are referenced: the “Little Lion‐Monkey” (*Saguinus oedipus*), Edwards (1751: 195, Plate 195); the “Little Black Monkey” (*Saguinus midas*), Edwards (1751: 196, Plate 196); the “Sanglin, or Cagui Minor” (*Callithrix jacchus*), Edwards (1758: 15, Plate 218); and the “Bush‐tailed Monkey” (*Sapajus apella*), Edwards (1764: 222, Plate 312). Interestingly, while the two primates from Edwards' London history appear on the same page as the “Bush‐tailed Monkey”, Linnaeus did not mention either the Spider Monkey or the brown monkey in any edition of *Systema Naturae*.

Unfortunately, the identity of Browne's and Edwards' primates remains uncertain, precluding definitive linkage to *Brachyteles*. Crucially, the absence of original specimens or illustrations renders direct comparison impossible, leaving their taxonomic affiliations speculative and irretrievable. There is, however, compelling evidence that can help us elucidate the routes of primate trafficking established to Jamaica. Sloane (1707: LV) documented the existence of a “private trade” between Jamaica and the “Spaniards of the West‐Indies”, which included the exchange of animals and goods originating from South American ports such as Santa Marta and Cartagena (located in what is now northwest Caribbean Colombia). Notably, Sloane also highlighted that many of these goods were shipped to England without significant political complications, facilitated by Jamaica's colonial status at the time.

Building on this line of evidence, we can examine Browne's (1756: 489) description of *Simia 3*, referred to as “The Tittee”. Browne describes this primate, noting that “the back and tail are of a clouded brown colour, and the belly whitish. The head is bare about the ears and eyes, but the hair grows in a narrow slip down the forehead”. This description aligns closely with the distinctive phenotype of the Colombian and Panamanian bare‐faced tamarins, which are classified within the oedipus group (Hershkovitz 1977; Rylands et al. 2016). Specifically, the traits described by Browne strongly suggest the Geoffroy's Tamarin (*Saguinus geoffroyi*), which is distributed in Central and eastern Panama extending into northwestern Colombia (Link et al. 2021). Given the relatively short distance between Jamaica and Colombian ports such as Santa Marta and Cartagena (approximately 850 km), it is plausible that the transport of medium‐ and large‐sized mammals occurred regularly. Supporting this idea, Browne's *Simia 1* is likely to be a brown species of *Alouatta*, perhaps *A. seniculus*, which was mentioned by Linné (1766: 37) to inhabit “Carthagenæ” ( = Cartagena). Finally, according to Gmelin (1788: 39) Browne's *Simia 4*—the *sake‐winkee*—would be a saki monkey, *Simia Pithecia* (=*Pithecia pithecia*). Nonetheless, the Latin description of this *Simia 4* does not match any known *Pithecia* species, and we were unable to identify it.

Considering all the evidence above, it is highly probable that a Colombian or Panamanian species of *Ateles* was also introduced to Jamaica and observed by Browne. Based on Browne's (1756) brief description of *Simia 2*, we propose that *Ateles hybridus* or a brown subspecies of *A. geoffroyi* are more plausible candidates for introduction to Jamaica rather than a *Brachyteles* specimen, given that the latter is endemic to the Brazilian Atlantic Forest, located nearly 5000 km south of Cartagena. This conclusion is further supported by the absence of evidence indicating direct or indirect trade between colonial Brazil and the island during that period. Additionally, the monkeys observed by Edwards in the streets of London were likely to have been shipped from Jamaica. In this context, Edwards' “black spider monkey” may correspond to the Colombian black spider monkey (*Ateles fusciceps rufiventris*), whereas the second, “the brown monkey”, aligns with the same potential candidates as Browne's *Simia 2*.

The first physical evidence of a *Brachyteles* individual emerged in 1808, when É. Geoffroy Saint‐Hilaire personally arranged to acquire zoological material from the Museu d'Ajuda during Napoleon's invasion of Portugal (Antunes 2011; Ceríaco 2021) (Figure 1). The precise origin of this specimen and the identity of its collector remain unclear. According to É. Geoffroy Saint‐Hilaire (1809: 92), D. Vandelli, the museum's director at the time, only revealed that the specimens originated from Brazil. Once at the Muséum National d'Histoire Naturelle (MNHN, Paris), É. Geoffroy Saint‐Hilaire (1809) used this material to provide a detailed description and illustration of his *Ateles arachnoides* (Figure 1). He also clarified that his 1806 mention of *A. arachnoides* in Annales du Muséum d'histoire naturelle (Vol. 7, p. 260) was a preliminary report, with the promise of a more comprehensive description to be presented in his subsequent work.

**Figure 1:**
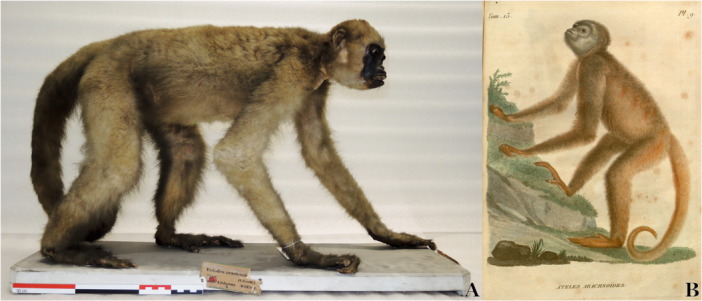
Claimed holotype of *Brachyteles arachnoides* (MNHN‐ZM‐2007‐1475) taken by É. Geoffroy Saint‐Hilaire in Lisbon, Portugal, 1808, and currently held at the Muséum national d'Histoire Naturelle–Paris (Photograph by Cécile Callou) (A). Original illustration of *Ateles arachnoides* É. Geoffroy Saint‐Hilaire (1809, Plate 9) (B).

A further complication in validating *Ateles arachnoides* É. Geoffroy Saint‐Hilaire 1806 arises from the disputed status of its purported type material. Both Serrano‐Villavicencio (2016) and Ceríaco (2021) identified specimen MNHN‐ZM‐2007‐1475 as the holotype of *Brachyteles arachnoides*. However, this designation conflicts with Article 72.4.1 of the ICZN, which requires that type material be demonstrably tied to the original description—either through direct physical evidence or explicit bibliographic reference. Critically, É. Geoffroy Saint‐Hilaire was unaware of this specimen's existence until 1808, 2 years after the name *Ateles arachnoides* first appeared (see Figure 2 for a chronological overview). Since MNHN‐ZM‐2007‐1475 cannot be definitively linked to É. Geoffroy Saint‐Hilaire 1806 description fails to meet the criteria for holotype designation under the ICZN. Consequently, this specimen cannot serve as the holotype of *Ateles arachnoides* É. Geoffroy Saint‐Hilaire 1806, casting further doubt on the validity of this taxonomic assignment.

**Figure 2:**
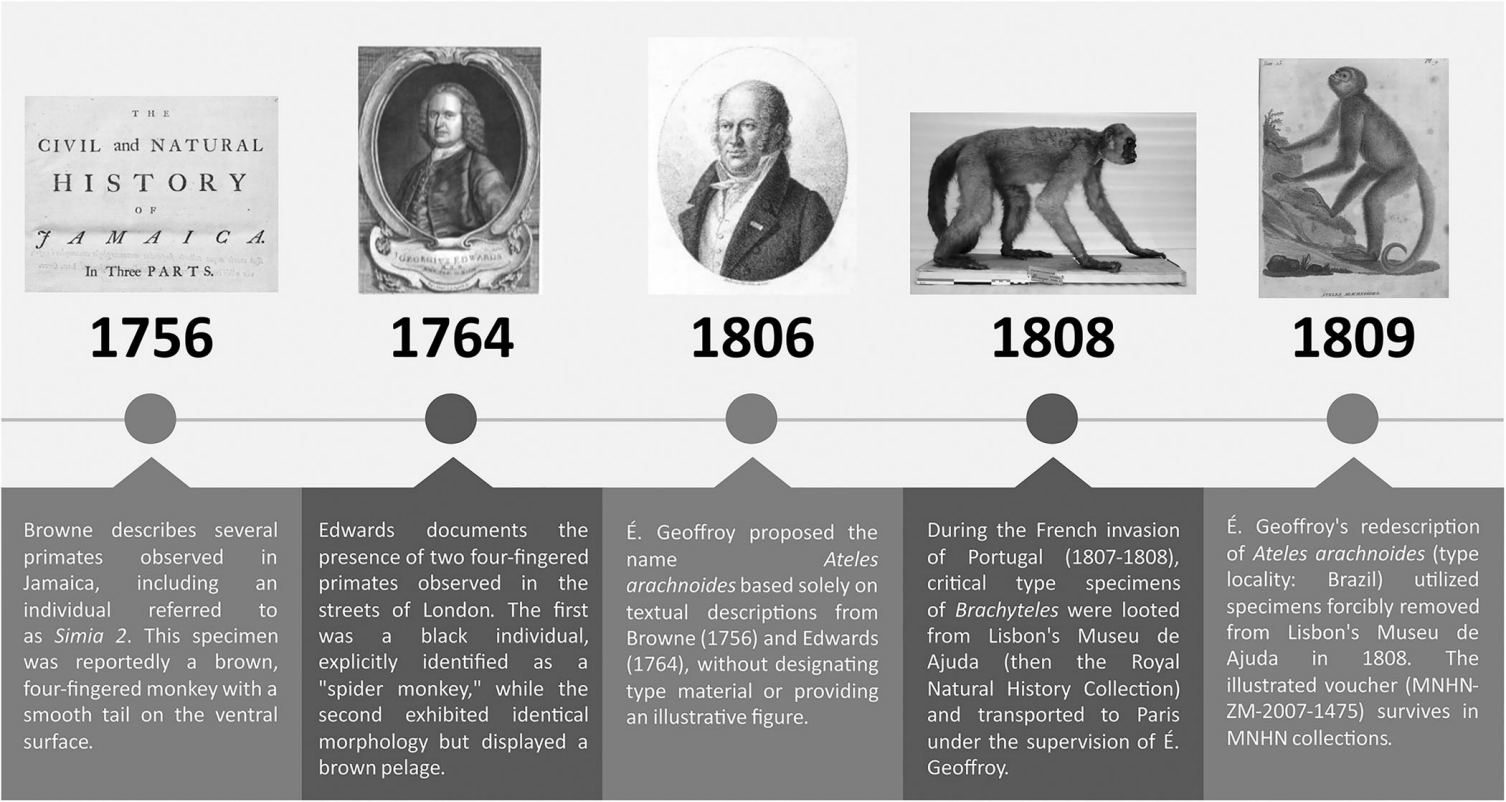
Timeline of the first landmark events of the history of *Brachyteles*, in which the appearance of the first individual of this species can be evidenced, which appeared 2 years after the early description of *Ateles arachnoides* in 1806.

In conclusion, since we cannot definitively establish that Browne's *Simia 2* and Edwards' “brown monkey” represent *Brachyteles* individuals, we propose that the name *Ateles arachnoides* É. Geoffroy Saint‐Hilaire 1806, should be regarded as a *nomen dubium*. We are not the first to challenge the identity of Browne's and Edwards' primates or their alleged connection to *Brachyteles*. Notably, Isidore Geoffroy Saint‐Hilaire (1829: 161) expressed skepticism about his father's sources, casting doubt on the accuracy of these earlier accounts: “*Tous les auteurs donnent comme synonyme le Singe mentionné par Edwards, Glan. d'Hist. nat., 5°. partie, et que l'on montroit à Londres sous le nom de Singe araignée, et le Singe à pelage brun dont parle Brown, Hist. de la Jamaïque. Ces deux indications doivent être considérées comme très‐douteuses*”. (= All authors give as synonyms the Monkey mentioned by Edwards, Glan. of Nat. History, 5th part, which was exhibited in London under the name of Spider Monkey and the Brown‐coated Monkey mentioned by Brown, Hist. of Jamaica. These two indications must be considered very doubtful.; our translation). By revising the authorship date, we address two critical issues: (1) we eliminate the longstanding confusion regarding the identity of Browne's *Simia 2* and Edwards' brown monkey, as well as the uncertainty of their association with *Brachyteles*; and (2) we establish, with certainty, a definitive connection between an existing specimen (MNHN‐ZM‐2007‐1475) and *Ateles arachnoides*. This specimen can be designated as the type (holotype by monotypy), supported by a comprehensive description and illustration of *A. arachnoides*. Finally, we provide an updated first‐use synonym for *Brachyteles arachnoides*.

***Brachyteles arachnoides*** (É. Geoffroy Saint‐Hilaire 1809).

*Ateles arachnoides* É. Geoffroy Saint‐Hilaire, 1806: 271; not determinable, a *nomen dubium*; based on Browne (1756: 489) *Simia 2* and Edwards (1764: 222) “brown long‐limbed four‐finger'd Monkey”, both unidentifiable atelids, probably an *Ateles* spp.

*Ateles arachnoides* É. Geoffroy Saint‐Hilaire (1809: 90); type locality “Brésil” (= Brazil).

*Simia arachnoides*: Humboldt 1812: 355; name combination; based on *Ateles arachnoides* of É. Geoffroy Saint‐Hilaire (1809: 90).

Atèle arachnoïde: Desmarest 1820: 75; vernacular, unavailable name; based on *Ateles arachnoides* of É. Geoffroy Saint‐Hilaire (1809, 90).

*Brachyteles macrotarsus* Spix 1823: 36. Part.

*Eriodes arachnoides*: I. Geoffroy Saint‐Hilaire 1829: 160; name combination; type locality “le Brésil”.

*Cebus arachnoides*: Fischer 1829: 38; name combination; based on *Ateles arachnoides* of É. Geoffroy Saint‐Hilaire (1809: 92).

*Brachyteles arachnoides*: Gray 1843: 10; type locality “Tropical America”; name combination; based on *Ateles arachnoides* of É. Geoffroy Saint‐Hilaire (1809: 92). First use of the current name combination.

*Brachyteleus arachnoides:* Elliot 1913: 50; unjustified emendation; plate III.

## Author Contributions

**José E. Serrano‐Villavicencio:** conceptualization (lead), data curation (equal), funding acquisition (equal), investigation (equal), visualization (equal), writing – original draft (lead), writing – review and editing (equal). **Joyce R. Prado:** funding acquisition (equal), investigation(equal), supervision (lead), visualization (equal), writing – review and editing (equal).

## Ethics Statement

Our research complies with the American Society of Primatologists' Principles for the Ethical Treatment of Nonhuman Primates. Ethical approval was not required since no live animals were used for research.

## Conflicts of Interest

The authors declare no conflicts of interest.

## Data Availability

Data sharing not applicable to this article as no data sets were generated or analyzed during the current study.
